# A native promoter–gene fusion created by CRISPR/Cas9‐mediated genomic deletion offers a transgene‐free method to drive oil accumulation in leaves

**DOI:** 10.1002/1873-3468.14365

**Published:** 2022-05-06

**Authors:** Rupam Kumar Bhunia, Guillaume N. Menard, Peter J. Eastmond

**Affiliations:** ^1^ 15552 Plant Sciences and the Bioeconomy Rothamsted Research Harpenden UK; ^2^ National Agri‐Food Biotechnology Institute (NABI) Mohali India

**Keywords:** CRISPR/Cas9, genome editing, promoter fusion, transcriptional gain‐of‐function, transgene‐free, plant oil metabolism

## Abstract

Achieving gain‐of‐function phenotypes without inserting foreign DNA is an important challenge for plant biotechnologists. Here, we show that a gene can be brought under the control of a promoter from an upstream gene by deleting the intervening genomic sequence using dual‐guide CRISPR/Cas9. We fuse the promoter of a nonessential photosynthesis‐related gene to *DIACYLGLYCEROL ACYLTRANSFERASE 2* (*DGAT2*) in the lipase‐deficient *sugar‐dependent 1* mutant of *Arabidopsis thaliana* to drive ectopic oil accumulation in leaves. *DGAT2* expression is enhanced more than 20‐fold and the triacylglycerol content increases by around 30‐fold. This deletion strategy offers a transgene‐free route to engineering traits that rely on transcriptional gain‐of‐function, such as producing high lipid forage to increase the productivity and sustainability of ruminant farming.

## Graphical abstract

Our study demonstrates a method to create a transcriptional gain‐of‐function without inserting foreign DNA into an organism. We bring a gene under the control of a promoter from an upstream gene by deleting the intervening genomic region using CRISPR/Cas9. We use this approach to engineer plant leaves to accumulate oil.

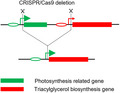

## Abbreviations

**Cas9**, CRISPR‐associated protein 9

**CRISPR**, clustered regularly interspaced short palindromic repeats

**DGAT**, diacylglycerol acyltransferase

**GMO**, genetically modified organism

**SDP1**, sugar‐dependent 1

**TAG**, triacylglycerol

Transcriptional gain‐of‐function is a basic tool in plant biotechnology, which traditionally relies on random or targeted insertion of foreign DNA to form a promoter‐gene fusion [1, 2]. The introduction of new genetic material has implications since the product is a genetically modified organism (GMO) [3]. There are many barriers to commercialization of GM crops and so it may be desirable to achieve transcriptional gain‐of‐function by other means, if possible. Genome editing technologies can be used to create single nucleotide substitutions, small insertions, deletions, and rearrangements without foreign DNA integration [4]. These changes are widely considered non‐GMO [3]. It is possible to modulate gene expression by editing *cis*‐regulatory elements, but the effects are generally subtle and can be hard to predict [5].

In this study, we tested whether the expression pattern of a native gene can be radically changed ‘to order’ by bringing it under the control of a promoter from an upstream gene, simply by deleting the intervening genomic sequence using CRISPR/Cas9 [4]. Lu et al. [6] recently reported a similar editing strategy that relies on creating large‐scale chromosomal inversions and duplications. We chose to engineer *Arabidopsis thaliana* leaves to accumulate storage oil (triacylglycerol) as a proof‐of‐concept. This is a synthetic trait with the potential to deliver a step change in crop oil yield [7, 8]. Even modest increases in leaf oil content, in the order of a few per cent of dry weight, can significantly increase livestock productivity and suppress enteric methane emissions in pasture‐based ruminant farming [9, 10].

## Materials and methods

### Plant material lines and growth conditions

Wild‐type *A. thaliana* ecotype Columbia (Col‐0) and *sdp1‐5* mutant seeds were described previously [11]. For plant growth experiments, the seeds were sterilized, applied to agar plates containing half‐strength Murashige and Skoog salts (pH 5.7) plus 1% (w/v) sucrose, and imbibed at 4 °C for 4 days. The plates were then transferred to a growth chamber set to 70% relative humidity [16 h light (22 °C)/8 h dark (18 °C); photosynthetic photon flux density = 250 µmol·m^−2^·s^−1^). After 2 weeks, seedlings were transplanted to 7‐cm^2^ pots containing moist Levington F2 compost and the plants were grown on in the growth chamber.

### Cloning and transformation

The CRISPR/Cas9 genome editing method we used to create deletions was adapted from [12]. DNA cassettes corresponding to 6728–14 724 bp of pEciCAS9‐Red (GenBank: KY489666) and 52–1325 bp of pEN‐2xChimera (GenBank: KY489664) were synthesized and cloned into pBinGlyRed and pUC57, respectively. Protospacer sequences were designed to target regions within the 5′‐untranslated regions (5′‐UTRs) of *DUG1* and *DGAT2* [13] using CRISPR‐PLANT [14]. They were then synthesized and cloned into pEN‐2xChimera using the BpiI and BsmBI restriction enzymes, respectively. The customized gRNAs were then transferred into pEciCAS9‐Red by Gateway single‐site LR recombination‐mediated cloning [12]. *A*. *thaliana* plants were transformed *via Agrobacterium tumefaciens*‐mediated floral dip [11].

### Selection of germinal deletions

Primary transformant (T_1_) seeds were selected using a Leica M205 fluorescence stereo microscope fitted with a DsRed filter (Leica Microsystems, Wetzlar, Germany). Around one hundred T_1_ plants were grown on soil and genomic DNA was extracted from 3‐week‐old plants. For PCR, genotyping [11] primer pairs DUG1P‐F and DGAT2G‐R were used (Table S1). Progeny of lines with somatic deletions were checked for a 3 : 1 segregation. DsRed‐negative T_2_ seeds of single‐locus lines were sown on soil and PCR genotyped for germinal deletion events [12]. Deletions were confirmed using Sanger DNA sequencing [12].

### Gene expression analysis and 5′‐RACE

For each sample, around 100 mg of tissue was ground in liquid nitrogen using a pestle and mortar. The Qiagen Plant RNeasy kit (Qiagen, Hilden, Germany) was used to extract RNA and DNase treat it following the manufacturer's protocol. The Superscript III kit (Thermo Fisher Scientific, Waltham, MA, USA) was used to produce the cDNA. cDNA samples were normalized, and quantitative PCR was performed with a Roche LightCycler 96 using the FastStart Essential DNA Green Master mix (Roche Molecular Systems, Pleasanton, CA, USA) following the procedure describe previously [15]. The primer pairs used for *DUG1* and *DGAT2* were QDUG1‐F & R and QDGAT2‐F & R, respectively (Table S1). The primer pairs used for the three reference genes (*UBIQUITIN5*, *ELONGATION FACTOR‐1α*, and *ACTIN8*) were described previously [15]. Data were analysed using the LightCycler 96 software and QBASE+ (Biogazelle, Gent, Belgium). Analysis of 5′ cDNA ends was performed with the 5′RACE system for Rapid Amplification of cDNA Ends (Thermo Fisher Scientific) using primers GSP1 and GSP2 (Table S1).

### Lipid analysis

Total lipids were extracted from homogenized freeze‐dried leaf tissue of plants that were 7 weeks old as described previously [11], and tripentadecanoic acid (15:0 TAG) was added to the homogenized tissue to act as an internal standard. A proportion of the total lipid extract was subjected directly to transmethylation, and the fatty acid methyl esters (FAMEs) were quantified by gas chromatography‐flame ionization detection (GC‐FID) with reference to the standard [11]. The remaining lipid extract was applied to silica thin layer chromatography plates, and neutral lipids were separated using a hexane : diethylether : acetic acid (70 : 30 : 1, v/v/v) solvent system. The lipids were visualized under UV light by staining with 0.05% (w/v) primuline in 80% (v/v) acetone, the TAG band was scraped from the plate and transmethylated, and the FAMEs were quantified by GC‐FID [11]. The total lipid content of seeds was determined by direct transmethylation and GC‐FID analysis of FAMEs [16].

### Microscopy

Lipid droplets were imaged *in situ* by laser scanning confocal microscopy using Nile red staining [10]. Nile red stock was made to a concentration of 10 mg·mL^−1^ in acetone and diluted to 10 µg·mL^−1^ in 0.01% (v/v) Triton x‐100 for a working concentration. Two detached leaves from each plant were vacuum infiltrated with Nile red solution, incubated for 5 h and 1 cm^2^ sections were mounted on slides in water and the abaxial surface imaged with a Zeiss LSM 980 with Airscan 2 (Zeiss, Jena, Germany). The images were captured in SR‐8Y mode using the C‐Apochromat 40×/1.2 W Korr FCS objective. Nile red was acquired with excitation 0.2% 541 nm Diode‐pumped solid state (DPSS) laser and chlorophyll was acquired with 0.4% 639 nm Diode laser. Emission for both channels was 527–735 nm to allow fast imaging to match the dynamics of lipid droplet movement. Spectra were obtained from one sample and used for linear unmixing of all samples to eliminate crosstalk between the Nile Red and chlorophyll. Acquisition of Z stacks encompassing the abaxial epidermal and spongey mesophyll cell layers were acquired between 55 and 60 µm depth (average Z stack 260 slices), and data are represented as orthogonal XY maximum projections of the Z stack.

## Results and Discussion

**Fig. 1:**
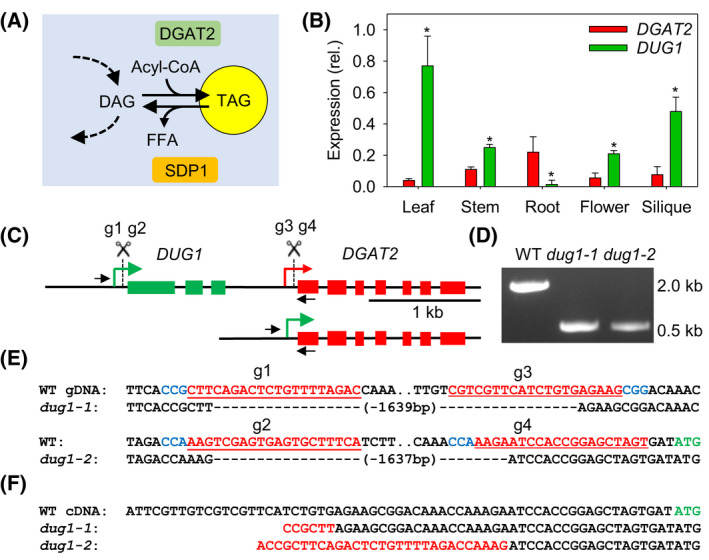
Selection of recipient and donor genes and creation of fusions using CRISPR/Cas9‐mediated genomic deletion. (A) Function of recipient gene *DGAT2* (and *SDP1*) in TAG metabolism. DAG, diacylglycerol; Acyl‐CoA, fatty acyl‐Coenzyme A; FFA, free fatty acid. (B) Quantitative RT‐PCR analysis of *DGAT2* and *DUG1* expression in various tissues. Values are presented as mean ± SE (*n* = 3) and are expressed relative to the geometric mean of three reference genes. Asterisks denote values significantly (*P* < 0.05) different from *DGAT2* (ANOVA + Tukey HSD test). (C) Genomic arrangement of *DGAT2* and *DUG1*. gRNA sites for CRISPR/Cas9 deletion are marked. (D) PCR performed on genomic DNA from homozygous *dug1‐1* and *dug1‐2* lines. Primer pairs are marked on C. (E) Genomic sequence spanning deletion sites. PAMs, gRNA sequences and start codon in blue, red, and green, respectively. (F) 5′‐UTRs of *DGAT2* determined by 5′‐RACE. *DUG1* sequence in red.

To test whether CRISPR/Cas9‐mediated genomic deletion can be used to bring a recipient gene that drives oil biosynthesis under the control of a donor promoter that is highly active in *A. thaliana* leaves, we selected *DIACYLGLYCEROL ACYLTRANSFERASE 2* (DGAT2, At3g51520) as the recipient [17]. DGAT2 is known to synthesize triacylglycerol (TAG) (Fig. 1A) and its overexpression is sufficient to drive ectopic oil production in leaves [17]. This oil accumulates particularly when turnover is impaired, for example by knocking out the lipase *SUGAR‐DEPENDENT1* (*SDP1*) [11] (Fig. 1A). Immediately upstream of *DGAT2* we found a donor gene we named *DGAT2 UPSTREAM GENE 1* (*DUG1,* At3g51510). *DUG1* encodes a chloroplast thylakoid‐associated protein of unknown function [18] that is much more strongly expressed in leaves than *DGAT2,* based on public microarray and RNA‐Seq data [19] (Fig. S1). We analysed *DGAT2* and *DUG1* transcript abundance in leaf, stem, root, flower, and silique tissues using quantitative RT‐PCR and confirmed that *DUG1* is around 20‐fold more strongly expressed in leaves than *DGAT2* (Fig. 1B).

The 5′‐UTRs of *DUG1* and *DGAT2* have previously been mapped using paired‐end analysis [13]. We designed multiple guide RNA (gRNA) that target within the 5′‐UTRs using CRISPR‐PLANT [14] (Fig. 1C, Fig. S2). We then cloned two gRNA pairs (g1–g3 and g2–g4) into a dual‐guide CRISPR/Cas9 binary vector derived from pEciCAS9‐Red [12] and we floral‐dip transformed the *sdp1‐5* mutant [10]. Following the procedure described by Durr et al. [12], we isolated two marker‐free homozygous ~ 1.6 kb germinal deletions, each created with a different gRNA pair, and named the mutants *dug1‐1* and *dug1‐2*. The deletion sites were characterized by genomic PCR and sequencing (Fig. 1D,E).

**Fig. 2:**
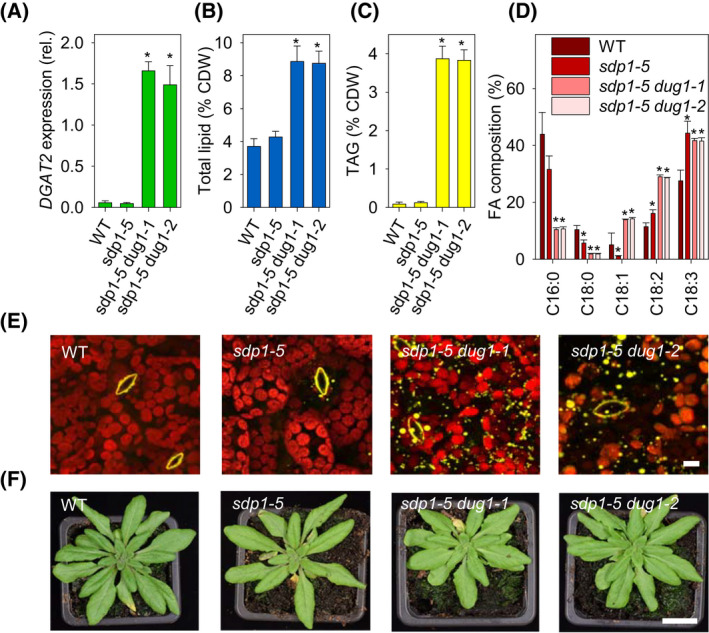
Effect of promoter fusion on lipid metabolism in rosette leaves of 7 weeks old plants. (A) Expression of *DGAT2* in leaves of *dug1‐1* and *dug1‐2* in the *sdp1‐5* background. (B) Total leaf lipid content and (C) TAG content, as percentage of cell dry weight (CDW). (D) Fatty acid composition of TAG. C16:0, palmitic acid; C18:0, stearic acid; C18:1, oleic acid; C18:2, linoleic acid; C18:3, linolenic acid. In A–D, data are presented as mean ± SE (*n* = 3) and asterisks denote values significantly (*P* < 0.05) different from WT (ANOVA + Tukey HSD test). (E) LSCM images of lipid droplets accumulating in leaves. Leaf sections were harvested ~ 6 h after dawn. Lipid droplets and guard cell cuticular ledges yellow (Nile red stained) and chloroplasts red (chlorophyll florescence). (F) Images of whole rosette plants. In E and F, the analyses were performed on multiple plants of each genotype (*n* = 3) and individual representative images are presented. Scale bars in E and F are 10 µm and 2 cm.

By performing 5′‐RACE on RNA from *dug1‐1* and *dug1‐2* leaves, we found that *DGAT2* transcripts possess chimeric 5′‐UTRs (Fig. 1F), which are consistent with initiation from the *DUG1* transcriptional start site [13]. Quantitative RT‐PCR analysis further showed that *DGAT2* transcript abundance is more than twenty‐fold higher in leaves of *sdp1‐5 dug1‐1* and *sdp1‐5 dug1‐2* than in either wild‐type (WT) or *sdp1‐5* (Fig. 2A). The modified expression pattern of *DGAT2* across leaf, stem, root, flower, and silique tissues also broadly mirrors that of *DUG1,* with the highest transcript abundance in leaves and the lowest in roots (Fig. S3).

Lipid analysis [11] showed that total lipid content of *sdp1‐5 dug1‐1* and *sdp1‐5 dug1‐2* leaves is around two‐fold higher than in WT or *sdp1‐5* (Fig. 2B), and that TAG content is around 30‐fold higher (Fig. 2C). The TAG contains more unsaturated fatty acids (Fig. 2D), which is consistent with the substrate preference of DGAT2 [17]. We also observed the accumulation of lipid droplets within leaf cells by laser scanning confocal microscopy (LSCM) using the fluorescent lipid stain Nile red [11] (Fig. 2E). The rosettes of 7 weeks old *sdp1‐5 dug1‐1* and *sdp1‐5 dug1‐2* plants are marginally smaller than those of either WT or *sdp1‐5*, but otherwise appear morphologically normal (Fig. 2F). The total lipid content of the seeds of the four genotypes is similar (Table S2). Germination and early seedling establishment are also similar when the seeds are sown on agar plates containing sucrose (Fig. S4). These data suggest that misexpression of *DGAT2* and loss of *DUG1* do not substantially alter *sdp1‐5* growth. However, because *A. thaliana* is an oilseed species and *sdp1‐5* is impaired in TAG hydrolysis [16], this genetic background does exhibit a reduced seedling establishment phenotype when its seeds are germinated on medium lacking an alternate carbon source, such as sucrose [16, 20].

## Conclusions

In this study, we show that a tissue‐specific transcriptional gain‐of‐function phenotype can be generated by creating a native promoter‐gene fusion using CRISPR/Cas9‐mediated genomic deletion. This approach relies on an appropriately expressed upstream donor gene lying in the right orientation and within deletion range. In our example the *DUG1* 5′‐UTR lies just 1.6 kb upstream of *DGAT2*. However, large deletions of tens or even hundreds of kb have been achieved in a variety of plants using CRISPR/Cas9 [4, 12, 21]. The approach is also contingent on deletion of the intervening gene(s) being tolerated. In this regard, the strategy could prove most durable in polyploids where gene redundancy is greatest [22]. Many crop plants and industrial microbial strains are polyploid. CRISPR/Cas9 is already widely used as a tool to knock out genes [4, 12, 21] and our study highlights that, when large deletions are created, it is important to consider the possibility that any phenotypes could be caused by misexpression of adjacent genes. Finally, the twofold increase in leaf total lipid content that we achieve here, without inserting foreign DNA, is likely sufficient to significantly enhance livestock productivity and reduce enteric methane emissions in pasture‐based ruminant farming systems, if translated to forage species. Evidence for this has been provided using a GMO approach to enhance oil content in perennial ryegrass (*Lolium perenne*) [9, 10].

## Author contributions

PJE conceived the idea and wrote the manuscript. RKB, GNM, and PJE conducted the experiments.

## Supporting information


**Fig. S1.** Developmental expression patterns of *DGAT2* and *DUG1*.
**Fig. S2.** gRNAs design.
**Fig. S3.** *DGAT2* expression in various tissues of *sdp1‐5 dug1‐1*.
**Fig. S4.** Seedling establishment.
**Table S1.** Primers used in study.
**Table S2.** Total lipid content of seeds.

## Data Availability

The data that support the findings of this study are available from the corresponding author (peter.eastmond@rothamsted.ac.uk) upon reasonable request.
