# Supplementary material for: A native promoter–gene fusion created by CRISPR/Cas9‐mediated genomic deletion offers a transgene‐free method to drive oil accumulation in leaves
Source: FEBS Lett. 2022 May 6;596(15):1865–70. doi: 10.1002/1873-3468.14365 (PMC9545981; doi:10.1002/1873-3468.14365)
Supplement: Supplementary file 1 — Fig. S1. Developmental expression patterns of DGAT2 and DUG1. Fig. S2. gRNAs design. Fig. S3. DGAT2 expression in various tissues of sdp1‐5 dug1‐1. Fig. S4. Seedling establishment. Table S1. Primers used in study. Table S2. Total lipid content of seeds. [file FEB2-596-1865-s001.docx]

**Supporting Information**

A native promoter–gene fusion created by CRISPR/Cas9-mediated genomic deletion offers a transgene-free method to drive oil accumulation in leaves

Rupam Kumar Bhunia^1, 2^, Guillaume N. Menard^1^, Peter J. Eastmond^1^

^1^Department of Plant Science, Rothamsted Research, Harpenden, Hertfordshire, AL5 2JQ, UK

^2^National Agri-Food Biotechnology Institute (NABI), Mohali, Punjab, 140306, India

**Fig. S1.** Developmental expression patterns of *DGAT2* and *DUG1*. Heat maps for *DGAT2* (At3g51520) and *DUG1* (At3g51510) gene expression derived from Affymetrix microarray data (Developmental Map) and RNA-Seq data (Klepikova Atlas) were generated by AtGenExpress eFP (bar.utoronto.ca/eplant/). Global Max Colour gradient setting was selected to allow quantitative comparison of gene expression between the two genes.


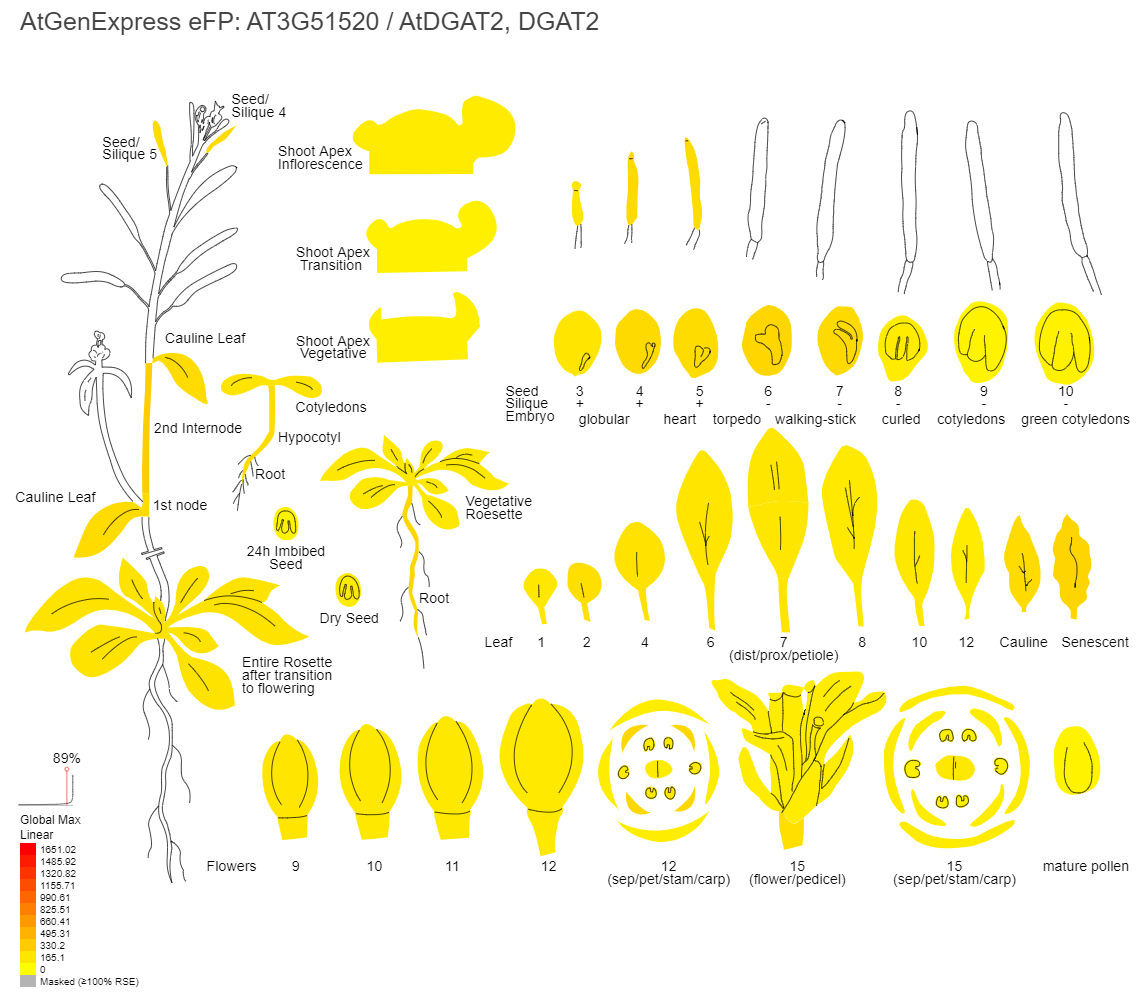

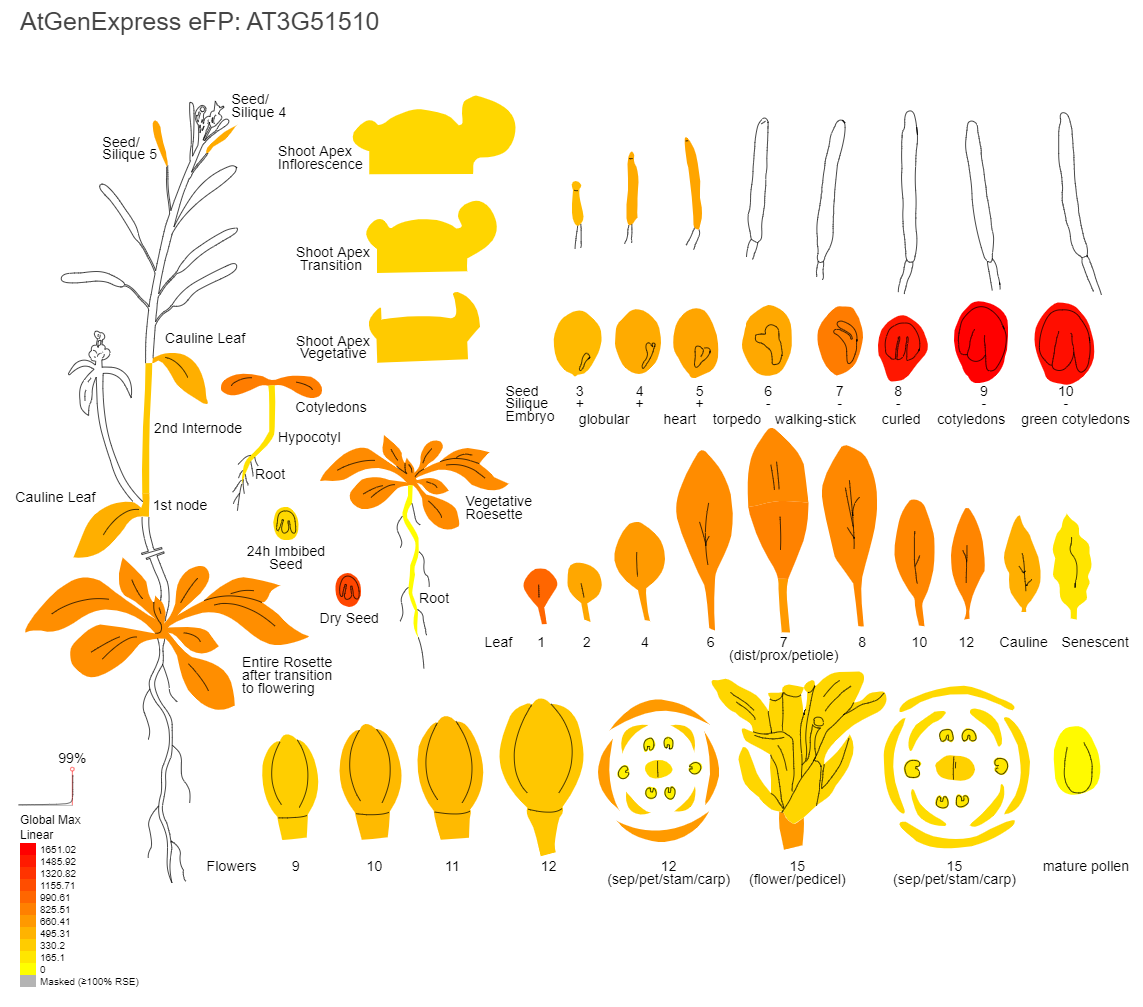

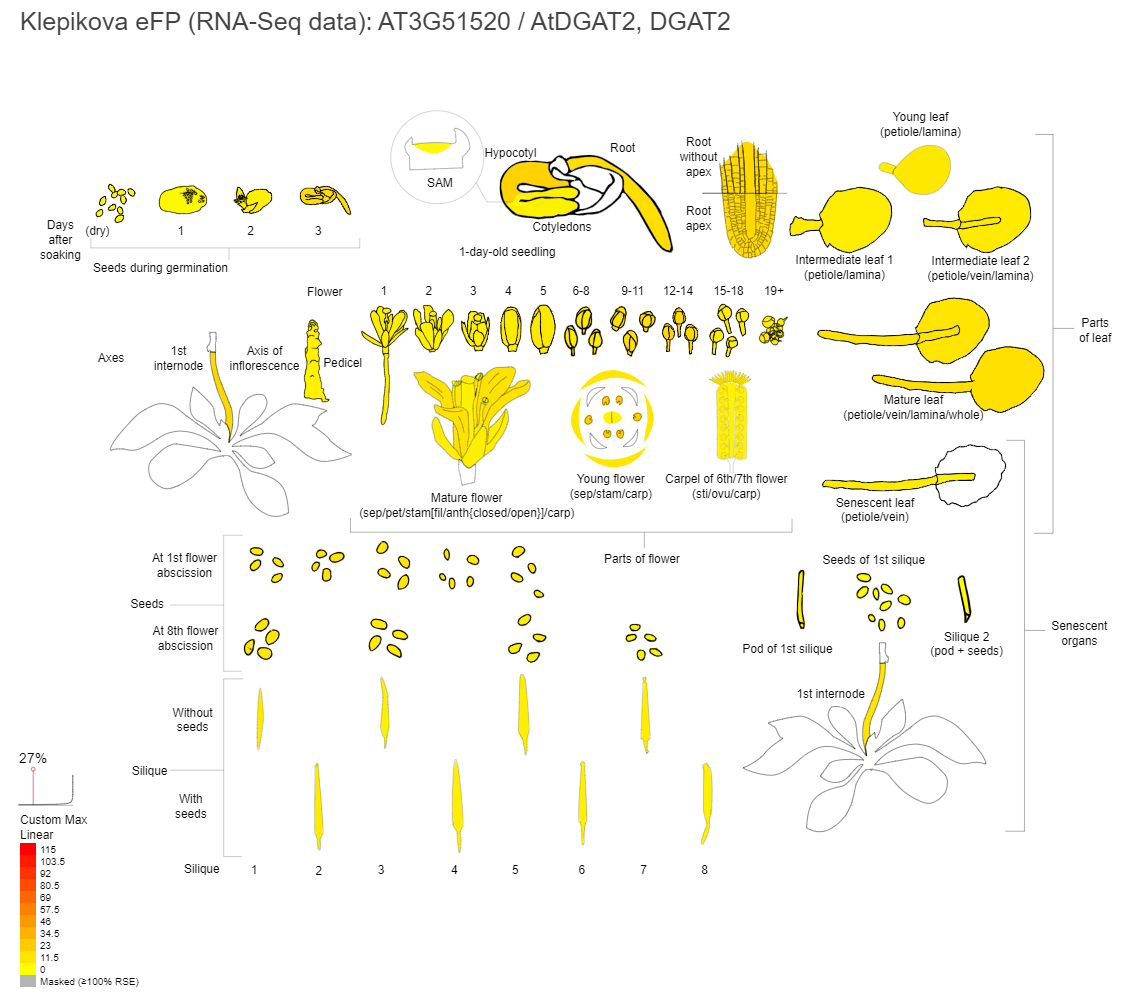

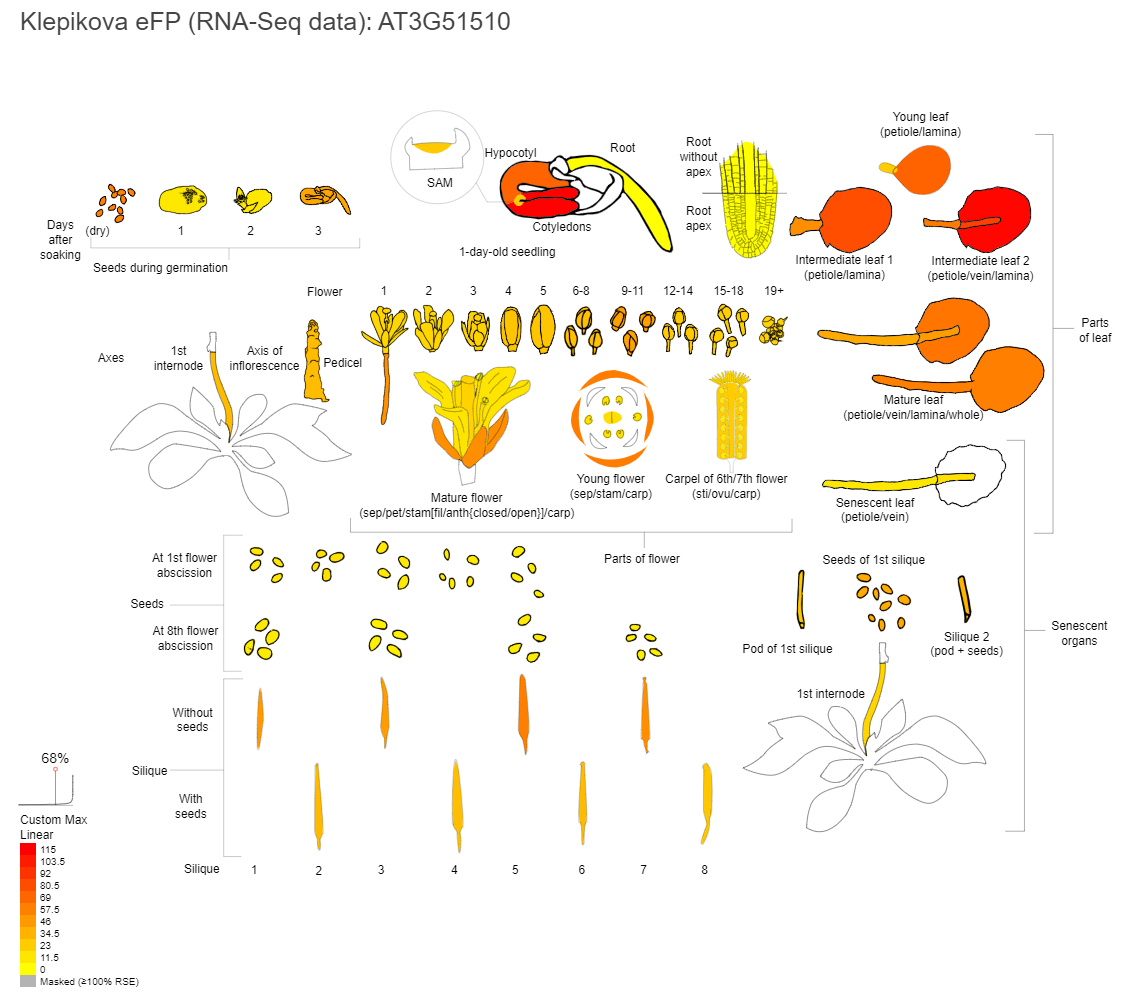


**Fig. S2.** gRNAs design. Untranslated sequences 5’ of the start codon were screened using CRISPR-PLANT. gRNA sites are underlined and PAMs are in bold. Double underlined nucleotides mark the putative start of transcription based on paired-end analysis.

AT3G51510 5’UTR

>Chr3:19108990..19109117 (+ strand) length=128

ACAGAATGATGATGGATTAGATATTTCTATTCAAAAACTATAACGTGTGGCTGCAAATCGATTCA**CCG**CTTCAGACTCTGTTTTAGA**CCA**AAGTCGAGTGAGTGCTTTCATCTTCTTCTTAAGCATCT

Class1.0 gRNA

SeqID minMM_GG minMM_AG Spacer seq (5'->3') PAM (5'->3')

Chr3:19109057-19109077:c 3 3 GTCTAAAACAGAGTCTGAAG **CGG**TGAATCG (gRNA1)

Chr3:19109079-19109099:c 4 4 TGAAAGCACTCACTCGACTT **TGG**TCTAAAA (gRNA2)

AT3G51520 5’UTR

>Chr3:19110596..19110738 (+ strand) length=143

AGTTAAAAGATTGGTTATTTGGGCTCTGCACTCAAGTGAGAGAGAAGATAGATAGATCTGAGTAGAATCTTCGATTCATTATTCGTTGTCGTCGTTCATCTGTGAGAAG**CGG**ACAAA**CCA**AAGAATCCACCGGAGCTAGTGAT

Class1.0 gRNA

SeqID minMM_GG minMM_AG Spacer seq (5'->3') PAM (5'->3')

Chr3:19110586-19110606 4 4 AAGTTGGGTAGTTAAAAGAT TGGTTATTTG

Chr3:19110684-19110704 5 4 CGTCGTTCATCTGTGAGAAG **CGG**ACAAACC (gRNA3)

Chr3:19110715-19110735:c 3 3 ACTAGCTCCGGTGGATTCTT **TGG**TTTGTCC (gRNA4)

Chr3:19110724-19110744:c 5 5 ACCCATATCACTAGCTCCGG TGGATTCTTT

Chr3:19110727-19110747:c 4 4 ACCACCCATATCACTAGCTC CGGTGGATTC

**
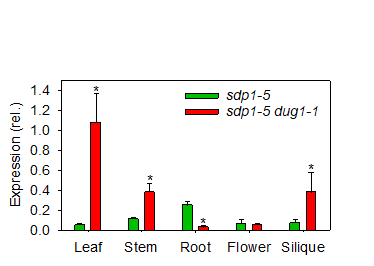
**

**Fig. S3.** *DGAT2* expression in various tissues of *sdp1-5 dug1-1*. Measurements were performed using quantitative RT-PCR. Values are presented as mean ± SE (n=3) and are expressed relative to the geometric mean of three reference genes. Asterisks denote values significantly (P < 0.05) different from *sdp1-5* (ANOVA + Tukey HSD test).

**
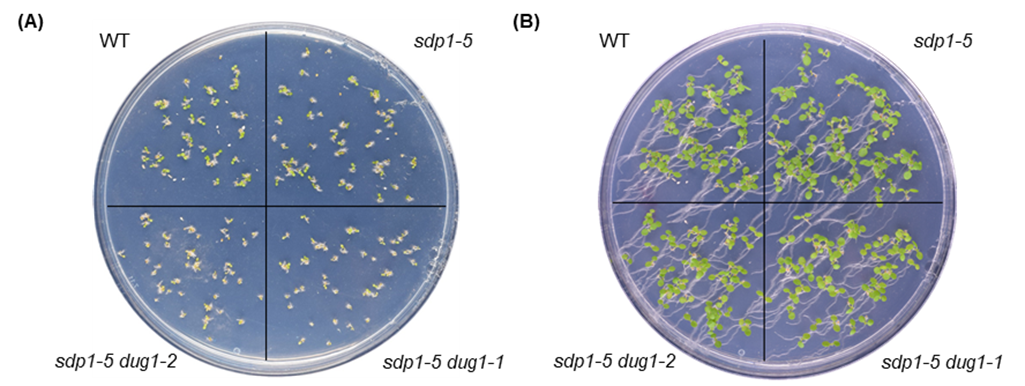
**

**Fig. S4.** Seedling establishment. Images of seedlings after (A) three days and (B) six days. The seedlings were grown on agar plates (9 cm diameter), containing half-strength Murashige and Skoog salts (pH 5.7) plus 1% (w/v) sucrose, as described in the Material and Methods section.

**Table S1.** Primers used in study.

**Genotyping**

DUG1P-F: 5’-TGTCGTTTATTTGCACCACG-3’

DGAT2G-R: 5’-AACAGAGAACAAGAGCGACG-3’

**Q-PCR**

QDUG1-F: 5’-TTCCTCATCCGCTCCG-3’

QDUG1-R: 5’-CAATGACTCCTGCGGC-3’

QDGAT2-F: 5’-TGGTGGAAGCCGGATT-3’

QDGAT2-R: 5’-CGGGACTTGTGCCTCT-3’

QACT8-F: 5’-GAATTACCCGACGGACA-3’

QACT8-R: 5’-ACGGTCTGCAATACCT-3’

QUBI5-F: 5’-GACGCTTCATCTCGTCC-3’

QUBI5-R: 5’-CCACAGGTTGCGTTAG-3’

QEF1α-F: 5’-TCCAGCTAAGGGTGCC-3’

QEF1α-R: 5’-GGTGGGTACTCGGAGA-3

**5’-RACE**

GSP1: 5’-CCAGGTACAAGAACACAACT-3’

GSP2: 5’-GAGCAACAACTCCAATCGGTAGCAC-3’

**Table S2.** Total lipid content of seeds.

| Genotype | Lipid content (% of CDW) |
| --- | --- |
| WT | 27.52 ±1.12 |
| *sdp1-5* | 28.15 ±0.58 |
| *sdp1-5 dug1-1* | 27.41 ±1.12 |
| *sdp1-5 dug1-2* | 28.25 ±1.67 |

Values are shown as a percentage of cell dry weight (CDW) and are the mean ± SE (n=3) of measurements on seed batches from plants of each genotype. The values are not significantly different (P > 0.05, ANOVA).
